# Work-Family Conflict and Primary and Secondary School Principals’ Work Engagement: A Moderated Mediation Model

**DOI:** 10.3389/fpsyg.2020.596385

**Published:** 2021-01-28

**Authors:** Zhongping Yang, Shisan Qi, Lianping Zeng, Xiaohong Han, Yun Pan

**Affiliations:** ^1^School of Psychology, Guizhou Normal University, Guiyang, China; ^2^School of Psychology, Inner Mongolia Normal University, Hohhot, China

**Keywords:** work-family conflict, job satisfaction, effective commitment, work engagement, primary and secondary school principal

## Abstract

With the development of positive psychology, work engagement has received widespread attention from researchers in the fields of positive organizational behavior and occupational health. Some studies have shown that work-family conflict has an important influence on individual behaviors and attitudes, but little research has studied the influence of work-family conflict on work engagement. The present study examined whether the relationship between work-family conflict and work engagement was mediated by job satisfaction, and whether the mediating role was moderated by affective commitment. We surveyed 358 Chinese primary and secondary school principals using the Work-Family Conflict Scale, Job Satisfaction Scale, Affective Commitment Scale, and Work Engagement Scale. The results revealed that there is a significant negative correlation between work-family conflict and primary and secondary school principals’ work engagement, and this relationship was partially mediated by job satisfaction. Moreover, affective commitment played a moderating role in the relationship between job satisfaction and work engagement. Specifically, the relationship between job satisfaction and work engagement was significant for primary and secondary school principals with high and low affective commitment. The current study contributes to a better understanding of the relationship between work-family conflict and work engagement.

## Introduction

Over the past decade, work engagement has received widespread attention in some areas (such as positive organizational behavior and occupational health; Guo et al., 2017). Work engagement is defined as a positive, fulfilling, and work-related state of mind (Schaufeli et al., 2002). There are three characteristics of work engagement: vigor, dedication, and absorption. Among them, vigor is shown by high levels of energy and willingness to invest effort in work, while dedication involves enthusiasm, pride, inspiration, and a sense of the significance of one’s work (Taghipour and Dezfuli, 2013). Absorption refers to concentrating completely and being happily engrossed in one’s work (Høigaard et al., 2012). Work engagement is an important indicator for measuring work attitude (Schaufeli and Bakker, 2004), and it brings some desirable outcomes for organizations and individuals (Kim et al., 2017). For example, Rich et al. (2010) pointed out that work engagement is closely related to many important organizational outcome variables, such as performance, organizational citizenship behavior, work withdrawal behavior, absence, and turnover intention. Many studies have found that employees with higher work engagement not only promote their ability and efficiency to work and gain a competitive advantage (Airila et al., 2012) but also significantly improve overall performance, profitability, organizational citizenship behavior, and customer satisfaction (Salanova et al., 2005; Xanthopoulou et al., 2007; Halbesleben et al., 2009). Given the benefits of high work engagement, further study on promoting employees’ work engagement is essential (Shantz et al., 2014).

Empirical research found that as the executors of school education, teachers’ work engagement can directly affect the healthy growth of students (Mao et al., 2019). Whether they can engage themselves to their work with high enthusiasm and full energy will not only have a direct impact on the development of a teacher’s career and students’ learning but also on the reform and development of education careers in China (Chen et al., 2018). Sheng (2006) also believed that teachers’ work engagement not only affect their own quality of life and professional growth but also affect the quality of school education and the healthy growth of students. Principals, as a special subgroup of teachers, are both educators and administrators, decision makers and managers; their work attitude and leadership style determine the school’s reputation, and their attitudes drive educational and leadership behaviors (Liang et al., 2016). Principles are professionals who perform the duties of school leadership and management (Ministry of Education of the People’s Republic of China, 2013). As the leader of school reform and development, principals are responsible for leading the development of schools and teachers, and promoting the overall development and personality development of students (Ministry of Education of the People’s Republic of China, 2013). The principal undertakes more things and plays multiple roles, not only responsible for teaching but also planning the development of the school. However, there is little research on the work engagement of primary and secondary school principals. Therefore, it is very necessary to explore the influencing factors and mechanisms of work engagement of primary and secondary school principals, which contributes to improve work efficiency and strengthen the cultivation and management of schools.

Work engagement is an important indicator for measuring work attitude (Schaufeli and Bakker, 2004). Studies have found that work-family conflict can influence individual behaviors and attitudes (Liu and You, 2019). It can be inferred that work-family conflict may affect individual’s work engagement. Role conflict theory holds that individuals have multiple roles, at work and in families, and need to assume different responsibilities and obligations in different environments (Liu and You, 2019). Meanwhile, following the conservation of resources theory (Hobfoll, 1989), individual energy and resources are limited, and when individuals use their resources in one field (e.g., work), there is a shortage of resources in another field (e.g., family), which increases the probability of conflicts. Conflict itself is a source of stress, which can cause strain, burnout, emotional exhaustion, and lack of work engagement (Mostert et al., 2011). Similarly, the study on special education teachers found that work-family conflict was negatively correlated with work engagement (Li, 2018). However, there is little empirical study on the impact of work-family conflict on work engagement (Liu and You, 2019). This indicates that the influencing path of work-family conflict on work engagement is not clear. Moreover, a study on 587 Chinese primary and secondary school principals showed that 44.4% of primary and secondary school principals work 50–59h a week, and 31.1% of them work more than 60h a week (Jiang and Zhao, 2014). This indicates that most of the primary and secondary school principals spend more time on their work, which leads to the reduction of family time and the conflict between work and family. Therefore, it is particularly important to explore the influencing path of work-family conflict on primary and secondary school principals’ work engagement.

Some studies have shown that certain work-related factors have an impact on work engagement through mediating variables (Liang et al., 2016). For instance, psychological state variables play a mediating role in the relationship between work-related characteristics and work engagement (May et al., 2011). Job satisfaction, the attitude of employees toward their work (Brief and Roberson, 1989), is an important psychological state related to work behavior intention and has an influence on employees’ work engagement levels (Wang and Yu, 2018). Empirical study has shown that job satisfaction is closely related to a positive work engagement (Pang and Wen, 2016), and job satisfaction plays a partially mediating role between family-work conflict and turnover intentions (Ma et al., 2017). Koscec (2003) found that the more satisfied employees are, the more engaged they are with the company. This indicates that there is a close relationship among work-family conflict, job satisfaction, and work engagement. However, there are few empirical studies on the role of job satisfaction in the relationship between work-family conflict and work engagement. Hence, this study examined job satisfaction as a potential mediator in the negative relationship between work-family conflict and work engagement.

Moreover, employees’ satisfaction with their work is strongly related to their commitment to the organization (Boles et al., 2007). Employee’s commitment is an important variable because a high level of commitment will bring beneficial outcomes to the organization (Tarkar et al., 2019). Empirical studies have found that the affective commitment [i.e., one of the three components of organizational commitment identified by Meyer and Allen (1991)] played a moderating role in the relationship between triggers of stress and job strain (Irving and Coleman, 2003). Specifically, the high affective commitment might strengthen the positive relationships between stressors and strain outcomes because highly committed employees are invested in and identified with organizations, and thus are more vulnerable to stressor experiences (Irving and Coleman, 2003; Liu et al., 2018). Employees with high affective commitment tend to have higher passion and enthusiasm for their organizations (Leung et al., 2004), and are willing to work harder to make more in-role and extra-role contributions to realize organizational goals (Alnıaçık et al., 2012). This indicates that affective commitment is a driving force and an important job resource (Eisenberger et al., 2010). Therefore, individuals who are satisfied with their work and show a commitment to the organization may contribute to invest in work.

In summary, the influence mechanism of work-family conflict on the work engagement of primary and secondary school principals is not clear. As the core figure of the school, the principal’s work attitude plays an important role in the development of school and basic education as well as teachers and students. Therefore, in order to provide theoretical support for guiding and supporting primary and secondary school principals to develop education more actively and effectively, this study takes primary and secondary school principals as the research object and mainly explores the influencing path of work-family conflict on their work engagement. The main purpose of the study is to investigate the relationship between work-family conflict and primary and secondary school principals’ work engagement, as well as the role of job satisfaction and affective commitment in the relationship between work-family conflict and work engagement, which is of great significance to improve their work efficiency, work level, and strengthen their cultivation and management.

## Theory and Hypotheses

### Work-Family Conflict and Work Engagement

Work-family conflict is defined as a form of inter-role conflict in which role pressures from the work and family domains are mutually incompatible (Greenhaus and Beutell, 1985). Work-family conflicts are bidirectional; work can cause family conflicts, and family can cause work conflicts (Grzywacz and Marks, 2000). The conservation of resources theory states that people strive to acquire and maintain resources that they value (Hobfoll, 1989). Since resources are scarce and people have to deal with a variety of work and family affairs, resources need to be allocated in a certain proportion and in a certain balance; thus, people tend to obtain more resources or maintain their original resource state (Li, 2018). However, when people do not have sufficient resources to understand or control the pressures they face, work-family conflicts easily result, which hinder people’s level of work engagement. Moreover, the role conflict theory states that if a person has more roles, the greater the possibility of pressure and burden of fulfilling these roles (Linzer et al., 2002), which in turn may reduce the degree of engagement to work. Many researches on the relationship between work-family conflict and work engagement mostly focus on enterprise employees and medical personnel, but pay less attention to primary and secondary school principals. For example, empirical studies on health care workers (Buonocore and Russo, 2013), nurse (Shao et al., 2015; Lang et al., 2019), hotel, and enterprise employees (Sun et al., 2011; Karatepe and Karadas, 2016; Wan et al., 2016; Liu and You, 2019; Zalewska, 2020) showed that work-family conflict was significantly correlated with work engagement; that is, a reduction in the former increased the level of work engagement of individuals. Moreover, the study on teachers also showed similar results: work-family conflict was negatively correlated with work engagement (Simbula et al., 2012; Li, 2018; Zeng et al., 2018). Based on this, we established our first hypothesis:

*Hypothesis* 1: Work-family conflict will relate negatively to work engagement.

### Work-Family Conflict and Job Satisfaction

The spillover theory of work-family conflict suggests that the emotions, attitudes, skills, and behaviors generated in the work and family domains will spill over from one domain (e.g., the work domain) to another (e.g., the family domain; Pryzby, 2005). Negative spillovers create conflict that depletes individuals’ time and energy, causing high levels of dissatisfaction (Zhou et al., 2011). Moreover, individuals with a high degree of work-family conflict constantly fight against the inner conflicts caused by the role responsibilities of work and family, causing more work depression (Hu and He, 2018), which causes less job satisfaction. Job satisfaction refers to an emotional state resulting from the appraisal of job (Locke, 1976). Some studies revealed that work-family conflict was negatively related to job satisfaction (Gao and Zhao, 2014; Armstrong et al., 2015; Baeriswyl et al., 2016; AlAzzam et al., 2017; Li and Wang, 2019) or that it led to low job satisfaction (Zhou et al., 2011). According to the conservation of resources theory, the loss of resources in one domain may lead to the loss of resources in other domains; when work interferes with family, the loss of resources is not only in the family domain but also likely to occur in the work domain, thereby reducing job satisfaction (Grandey and Cropanzano, 1999). Based on this, we established our second hypothesis:

*Hypothesis* 2: Work-family conflict will relate negatively to job satisfaction.

### The Mediating Role of Job Satisfaction

Most current research investigates the direct impact of work-family conflict on its outcome variables such as work attitudes and behavioral tendencies of employees, while less attention is paid to the internal mechanisms of work-family conflicts that produce these effects, so the intermediate processes of these effects are still in a “black box” to a certain extent (Gao and Zhao, 2014). At present, many studies have paid attention to the mediating role of job satisfaction, such as work-family conflict indirectly affects employee’s job performance by job satisfaction (Aminah, 2008), and job satisfaction plays a mediating role in the relationship between work-family conflict and physicians’ turnover intention (Lu et al., 2017), the relationship between bidirectional work-family conflict and turnover intention (Chen et al., 2014), and the relationship between family-to-work conflict and job performance (Ma et al., 2017). Therefore, job satisfaction may play a mediating role in the relationship between work-family conflict and its outcome variables.

According to resource conservation theory (Hobfoll, 1989), individuals maintain and acquire resources. However, due to the limited resources, when there is conflict between work and family, if the depleted resources are not supplemented in time, it will have a negative impact on people’s work attitude and behavior tendency (Gao and Zhao, 2014). Studies have found that job satisfaction is one of the key attitudes associated with work engagement (Saks, 2006; Simpson, 2008). In other words, if people are satisfied with their jobs, their engagement may increase. Conversely, if employees are not satisfied in an organization, their level of work engagement may decrease. This has been demonstrated in empirical studies that showed that job satisfaction is related to employee engagement (Taghipour and Dezfuli, 2013; Huang et al., 2016; Wang et al., 2017), and job satisfaction is an antecedent of employee engagement (Abraham, 2012; Bellani et al., 2017; Yalabik et al., 2017). Studies with teachers also showed similar results, such as kindergarten teachers with higher job satisfaction can find their own values and points of interest in their work, show themselves in roles, and are more likely to perceive positive factors in their work and generate higher work engagement than teachers with lower job satisfaction (Houston et al., 2012); there is positive correlations between health science teachers’ job satisfaction and work engagement (Park and Johnson, 2019). Furthermore, some studies have shown that individuals increase professional engagement when they frequently experience positive emotions and satisfaction in an organization (such as school or workplace; Reschly et al., 2008; Simpson, 2009). These studies indicate that there is a strong correlation between job satisfaction and work engagement. Therefore, job satisfaction has an indirect role in the relationship between work-family conflict and its outcome variables, which leads to our third hypothesis:

*Hypothesis* 3: Job satisfaction plays a mediating role in the relationship between work-family conflict and work engagement.

### The Moderating Role of Affective Commitment

Organizational commitment is an important study topic in the field of organizational behavior (Morrow, 2011). Organizational commitment refers to a mental state that reflects the relationship between employees and the organization and affects the decision of whether employees remain in the organization (Allen and Meyer, 1990) and includes three components, namely: affective commitment, continuance commitment, and normative commitment. Among them, affective commitment refers to employees’ emotional attachment to, identification with, and involvement in their organizations; normative commitment refers to a sense of obligation to the organization; continuance commitment refers to the perceived cost of leaving the organization. Although the three components generate positive effects for the individual and the company alike, affective commitment represents more significant benefits in terms of job satisfaction, reduced possibility of turnover, and worker stress (Meyer et al., 2002). Moreover, affective commitment is considered to have the strongest predictive power for employees’ job outcomes (Wright and Bonett, 2002; Leung et al., 2008; Cao et al., 2020), and to be one of the most important and core component of organizational commitment (Zhou et al., 2016; Guo et al., 2019). Previous studies have showed that high levels of affective commitment were positively correlated with individual-related outcomes (e.g., work efficiency, work enthusiasm, and job performance; Namasivayam and Zhao, 2007; Leung et al., 2008; Cao et al., 2020). This suggests that affective commitment is a key predictor of employee’s behavior (Cao et al., 2020).

Affective commitment is regarded as a moderator, which might buffer or enhance the relationship between variables (Meyer and Maltin, 2010). For example, some studies showed that affective commitment may enhance the relationship between stressors and strain outcomes (Irving and Coleman, 2003; Meyer and Maltin, 2010), autonomy and engagement (Dominguez et al., 2020), workplace incivility and burnout (Liu et al., 2018). A recent study of special employment center employee also showed that affective commitment significantly moderated the relationship between satisfaction with coworkers and supervisors and turnover intention (Romeo et al., 2020). Those studies indicate that the affective commitment may play a moderating role in the relationship between job-related variables and its outcome variables. A study found that employees with high affective commitment are more engaged in their work roles (Mayer and Schoorman, 1992). Employees with high affective commitment are driven by positive emotions; they not only identify strongly with the goals and values of the organization but also have a high sense of belonging in and attached to the organization and are willing to fulfill expectations and make extra work efforts (Sun et al., 2015). This indicates that affective commitment may contribute to improve the level of employees’ work engagement. Moreover, following the conservation of resources theory, individuals who are highly loyal and attached to the organization are more willing to engage resources (such as time and energy) to achieve the goals of the organization because they do not think that working for long hours or sustained work pressure is a depressing thing, and their job satisfaction has not decreased (Cao et al., 2020). Based on this premise, we formulated our final hypothesis:

*Hypothesis* 4: Affective commitment will moderate the mediated relationship between work-family conflict and work engagement *via* job satisfaction. Specifically, the relationship between job satisfaction and work engagement will be stronger when affective commitment is high compared to when it is low.

**Figure 1:**
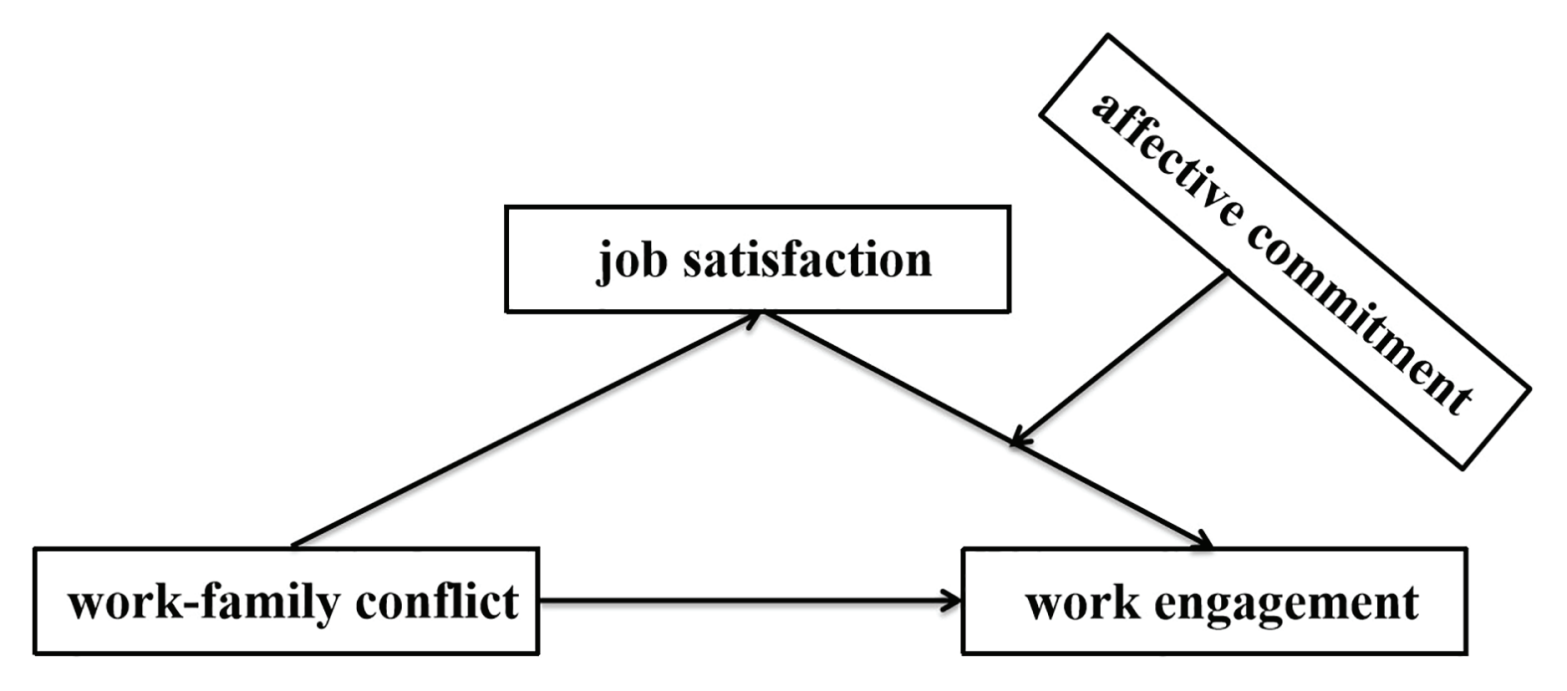
The hypothesized model of moderated mediation.

We established a moderated mediation model for the relationship between work-family conflict and work engagement (Figure 1).

## Materials and Methods

### Participants and Procedure

Participants in the current study were principals at Chinese primary and secondary schools. The study involved human participants and was reviewed and approved by the morality and ethics committee of the School of Psychology, Guizhou Normal University. The participants provided oral informed consent to participate in this study. Primary and secondary school principals were recruited using convenience sampling methods in the Liaoning and Guizhou provinces of China. Through the local education bureau, we contacted school principals and explained the purpose and requirements of the questionnaire. A total of 380 questionnaires were distributed and returned; 358 (94.21%) participants provided valid responses. The invalid data mainly included instances where participants did not answer carefully, for example, they provided the same response to all items consistently. Among the participants, 252 were male (70.39%), 100 were female (27.93%), and 6 (1.68%) did not provide data regarding gender. Twenty-five participants were between 25 and 34years old (6.98%), 192 participants were 35 to 44years old (53.63%), 125 participants were 45 to 54years old (34.92%), 12 were above 55 (3.35%), and 4 participants (1.12%) did not respond. A slight majority (185 participants) of participants had undergraduate degrees (51.68%), while 97 participants had less than an undergraduate degree (27.09%) and 70 participants (19.55%) had a postgraduate degree or higher. Six participants (1.68%) did not provide educational data.

### Measures

#### Work-Family Conflict Scale

We assessed work-family conflict with the Work-Family Balance Scale developed by Grzywacz and Marks (2000). The Chinese version of the Work-Family Balance Scale was revised by Zeng and Yan (2013). This study used only the eight-item Work-Family Conflict sub-scale, with items such as “Your job makes you feel too tired to do the things that need attention at home” and “Personal or family worries and problems distract you when you are at work.” The sub-scale consists of two dimensions: work-to-family conflict and family-to-work conflict. Participants rated their levels of work-family conflict on a five-point Likert scale (1=*never*, 5=*always*). The higher the score is, the higher the level of work-family conflict. In this study, the Cronbach’s alpha for the scale was 0.76.

#### Job Satisfaction Scale

We assessed job satisfaction with the three-item Job Satisfaction Subscale from the Michigan Organizational Assessment Questionnaire (Cammann et al., 1979). The Chinese version of the Job Satisfaction Subscale of the Michigan Organizational Assessment Questionnaire was revised by Liu et al. (2007). Example items include “Overall, I am very satisfied with my work” and “In general, I do not like my work.” Participants rated their levels of job satisfaction on a five-point Likert scale (1=*strongly disagree*, 5=*strongly agree*). One item in the scale is reverse scored. The higher the score of the participant is, the higher the level of job satisfaction. In this study, the Cronbach’s alpha for the scale was 0.81.

#### Affective Commitment Scale

We assessed affective commitment with the Organizational Commitment Scale developed by Allen and Meyer (1990). The Chinese version of the Organizational Commitment Scale was revised by Li et al. (2006). This study used only the eight-item Affective Commitment sub-scale. Due to the exploratory factor, analysis showed that the factor load value of one item was 0.45 in the original scale (Allen and Meyer, 1990). According to the standard that factor loadings in the structure matrix should be greater than 0.5, this item was not included in this study. Example items include “I enjoy discussing my organization with people outside it” and “This organization has a great deal of personal meaning for me.” Participants rated their levels of affective commitment on a five-point Likert scale (1=*strongly disagree*, 5=*strongly agree*). Two items are reverse scored. The higher the score is, the higher the level of affective commitment. In this study, the Cronbach’s alpha for the scale was 0.82.

#### Work Engagement Scale

We assessed work engagement with the 17-item Utrecht Work Engagement Scale (UWES) developed by Schaufeli et al. (2002). The Chinese version of the UWES was revised by Zhang and Gan (2005). Example items include “At my work I always persevere, even when things do not go well” and “I find the work that I do full of meaning and purpose.” The scale includes three dimensions: vigor, dedication, and absorption. Participants rate their levels of work engagement on a five-point Likert scale (1=*disagree*, 5=*agree*; Zeng et al., 2018). The higher the score is, the higher the level of work engagement. In this study, the Cronbach’s alpha for the scale was 0.95.

#### Control Variables

According to previous studies, employees of different sexes, ages, and education may have different working and personal resources (Xanthopoulou et al., 2007). These resources directly affect people’s experience of job roles and the level of work engagement (Seibert et al., 2011; Bakker and Demerouti, 2017). Therefore, gender, age, and education were used as control variables in the current study. Based on a previous study (Li et al., 2020), we coded the control variables. In terms of gender, male was coded as “1” and female as “2.” The age category 25–34years old was coded as “1,” 35–44 as “2,” 45–54 as “3,” and above 55 as “4.” For education, less than an undergraduate degree was coded as “1,” undergraduate degree as “2,” and postgraduate degree or higher as “3.”

### Data Analysis

The data were analyzed using the following procedures: First, the Jamovi software package (Navarro and Foxcroft, 2018) was used to perform the confirmatory factor analysis to examine the construct validity of the key variables. Second, the common method bias problem was examined using the Harman’s single-factor test. Third, descriptive statistics and Pearson correlations were performed to assess relationships among key variables. Fourth, model 4 of the SPSS PROCESS macro (Hayes, 2013) was used to examine the mediating roles of job satisfaction in the relationship between work-family conflict and work engagement based on the bias-corrected percentile bootstrap method (5,000 samples). Further, model 14 of the SPSS PROCESS macro was used to examine the role of affective commitment in the moderated mediation model using the same method as described for model 4.

## Results

### Common Method Bias Test

A common method bias test was conducted using the Harman’s single-factor test (Podsakoff et al., 2003). The results showed that the KMO value was 0.98 (*p*<0.001), indicating that the scales are suitable for factor analysis. There were eight factors with eigenvalues greater than 1, and the first factor explained a variance of 34.63%, which did not reach the critical criterion of 40%. Therefore, the influence of common method bias is not considered to be great in this study.

### Confirmatory Factor Analysis

To test the construct validity of the key variables, we conducted confirmatory factor analysis (CFA) using Jamovi (Navarro and Foxcroft, 2018). According to Wang et al.’s (2005) method for testing the construct distinctiveness of study variables, we compared a one-factor model (Model 1), a two-factor model (Model 2), a three-factor model (Model 3), and a four-factor model (Model 4). In Model 1, we loaded work-family balance, job satisfaction, affective commitment, and work engagement items on one factor. In Model 2, based on previous studies (Zeng and Yan, 2013; Hu and Wang, 2014), we loaded work-family conflict and job satisfaction items on one factor and work engagement and affective commitment items on another factor. In Model 3, based on a previous study (Hu and Wang, 2014; Zeng et al., 2018), we loaded work-family conflict and work engagement items on one factor, and job satisfaction and affective commitment on another factor, respectively. In Model 4, we treated the four constructs (work-family balance, job satisfaction, affective commitment, work engagement) as four independent factors. The result showed that Model 4 fit the data better than the other models and indicated good construct validity (see Table 1).

**Table 1:** Confirmatory factor analysis to assess construct validity.

Model	Factor loaded	χ^2^	df	χ^2^/df	CFI	TLI	RMSEA	Δχ^2^	Δdf	contrast
Model 1	One factor: WFC+JS+WE+AC	588	90	6.53	0.85	0.82	0.12			
Model 2	Two factors: WFC+JS, WE+AC	573	89	6.44	0.85	0.82	0.12	15	1	√
Model 3	Three factors: WFC+WE, JS, AC	439	87	5.05	0.89	0.87	0.11	24	2	√
Model 4	Four factors: WFC, JS, WE, AC	343	84	4.08	0.92	0.90	0.09	96	2	√

WFC, work-family conflict; JS, job satisfaction; WE, work engagement; AC, affective commitment; +, the combination of two factors into one factor. √, the current model is superior to the base model and the contrast is significant.

### Descriptive Statistics and Correlation Analysis

As shown in Table 2, the skewness and kurtosis values did not reach the critical criterion (i.e., skewness<|2.0| and kurtosis<|7.0|; Hancock and Mueller, 2010), this indicated that all variables were normally distributed, and an analysis of the correlations revealed that work-family conflict was negatively related to work engagement (*r*=−0.21, *p*<0.01) and job satisfaction (*r*=−0.14, *p*<0.01), supporting hypotheses 1 and 2. However, there was no significant correlation between work-family conflict and affective commitment (*r*=0.03, *p*>0.05). The results also indicated that job satisfaction was positively related to work engagement (*r*=0.50, *p*<0.01) and affective commitment (*r*=0.32, *p*<0.01). In addition, affective commitment was positively related to work engagement (*r*=0.28, *p*<0.01).

**Table 2:** Descriptive statistics and correlations between study variables.

Variable	*M*	*SD*	Skewness	Kurtosis	1	2	3	4
1. Work-family conflict	2.51	0.86	0.11	−0.72	-			
2. Job satisfaction	3.29	0.63	−0.59	1.36	−0.14^*^	-		
3. Work engagement	3.77	0.96	−1.22	1.05	−0.21^**^	0.50^**^	-	
4. Affective commitment	3.14	0.50	0.05	1.39	0.03	0.32^**^	0.28^**^	-

* *p*<0.05

** *p*<0.01.

### The Mediation Role of Job Satisfaction

The first mediating model examined whether job satisfaction mediated the relationship between work-family conflict and work engagement. To examine the mediating role, we adopted the SPSS PROCESS Macros Model 4 (Hayes, 2013). This bootstrapping technique is considered the most powerful and reasonable to obtain confidence intervals for indirect effects (Williams and Mackinnon, 2008). Mediation was established when the indirect effect was significant, and the confidence intervals did not contain a zero. As shown in Table 3, after controlling for participants’ demographics (e.g., gender, age, and educational level), the results showed that work-family conflict was negatively associated with work engagement (Model 1: *β*=−0.31, *t*=−5.57, *p*<0.001) and job satisfaction (Model 2: *β*=−0.15, *t*=−3.84, *p*<0.001). Moreover, job satisfaction was positively associated with work engagement (Model 3: *β*=0.57, *t*=7.81, *p*<0.001), and the direct association between work-family conflict and work engagement remained significant [Model 3: *β*=−0.22, *t*=−4.31, 95% CI=(−0.33, −0.12)]. The bias-corrected percentile bootstrap analyses showed that job satisfaction had a significant partially mediating role in the relationship between work-family conflict and work engagement (indirect effect=−0.08, 95% CI=[−0.13, −0.03]), supporting hypothesis 3.

**Table 3:** Mediating effect of job satisfaction in the relationship between work-family conflict and work engagement.

Dependent variable	Model 1 (work engagement)			Model 2 (job satisfaction)			Model 3 (work engagement)		
	*β*	*t*	95%CI	*β*	*t*	95%CI	*β*	*t*	95%CI
Gender	−0.44^***^	−4.17	[−0.65, −0.23]	−0.29^***^	−4.00	[−0.44, −0.15]	−0.28^**^	−2.75	[−0.47, −0.08]
Age	0.03	0.37	[−0.11, 0.16]	0.05	1.02	[−0.05, 0.15]	−0.00	−0.04	[−0.13, 0.13]
Educational level	−0.51^***^	−7.20	[−0.64, −0.37]	−0.24^***^	−4.83	[−0.33, −0.14]	−0.37^***^	−5.56	[−0.50, −0.24]
Work-family conflict	−0.31^***^	−5.57	[−0.42, −0.20]	−0.15^***^	−3.84	[−0.22, −0.07]	−0.22^***^	−4.31	[−0.33, −0.12]
Job satisfaction							0.57^***^	7.81	[0.43, 0.71]
*R*^2^	0.24			0.15			0.36		
*F*	25.81^***^			14.38^***^			36.58^***^		

CI, confidence interval; it represents significance when the confidence intervals did not contain zero.

** *p*<0.01

*** *p*<0.001.

### The Moderating Role of Affective Commitment

**Figure 2:**
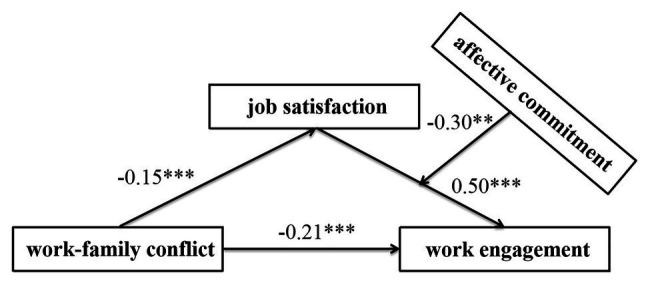
Path coefficients of the moderated mediation model. JS, job satisfaction; AC, affective commitment. ^**^p<0.01, ^***^p<0.001.

We used the SPSS PROCESS Macros Model 14 (Hayes, 2013) to examine the moderating role of affective commitment. Overall testing models are presented in Figure 2, and the specific indirect effects are presented in Table 4. As outlined by Hayes (2013), the conditional indirect effect was established when the interactions between job satisfaction and affective commitment were significant, and the bootstrapping confidence intervals did not contain zero. As shown in Figure 2 and Table 4, affective commitment significantly moderated the indirect effect of work-family conflict on work engagement *via* job satisfaction. The results showed that job satisfaction was positively associated with work engagement (*β*=0.50, *p*<0.001), and the product (interaction term) of job satisfaction and affective commitment had a significant role on work engagement [*β*=−0.30, 95% CI=(0.51, −0.08)]. In addition, the direct effect of work-family conflict on work engagement was −0.21 [BootSE=−0.05, 95% CI=(−0.32, −0.11)], and the index of the moderated mediation was 0.04 [BootSE=0.02, 95% CI=(0.01, 0.09)].

**Table 4:** Results of affective commitment moderate the mediation process.

Dependent variable	Model 1 (job satisfaction)			Model 2 (work engagement)		
	*β*	*t*	95%CI	*β*	*t*	95%CI
Gender	−0.29^***^	−3.99	[−0.44, −0.15]	−0.24^*^	−2.42	[−0.43, −0.04]
Age	0.05	0.98	[−0.05, 0.15]	−0.02	−0.23	[−0.14, 0.11]
Education	−0.23^***^	−4.78	[−0.33, −0.14]	−0.35^***^	−5.30	[−0.48, −0.22]
Work-family conflict	−0.15^***^	−3.84	[−0.22, −0.07]	−0.21^***^	−4.17	[−0.32, −0.11]
Job satisfaction				0.50^***^	6.74	[0.36, 0.65]
Affective commitment				0.29^**^	3.19	[0.11, 0.46]
Job satisfaction×affective commitment				−0.30^**^	−2.75	[−0.51, −0.08]
*R*^2^	0.15			0.39		
*F*	14.25^***^			29.74^***^		
**Moderate mediation index**	**BootSE**			**95% CI**		
0.04	0.02			[0.01, 0.09]		

×, interaction item.

* *p*<0.05

** *p*<0.01

*** *p*<0.001.

**Figure 3:**
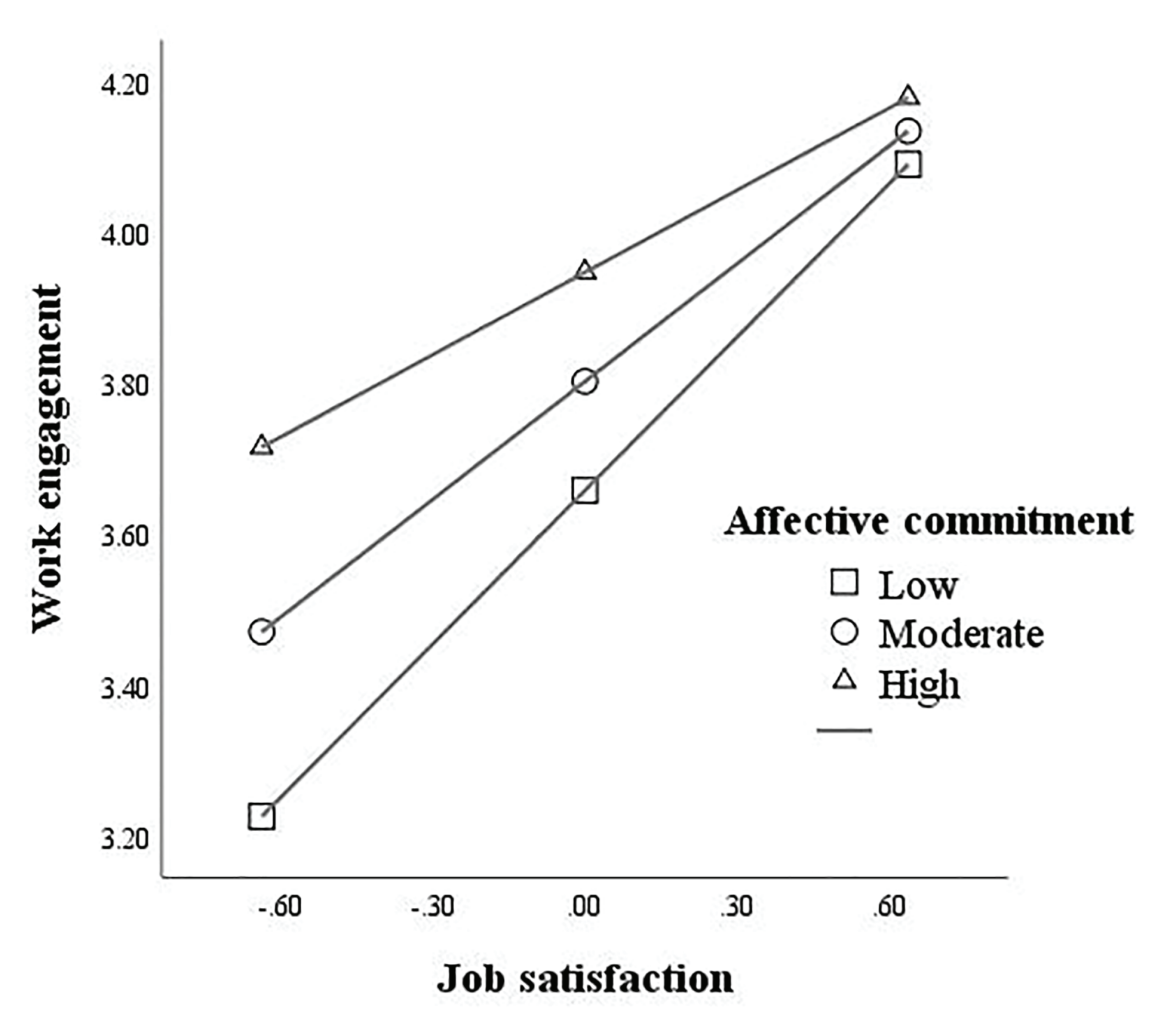
Simple slopes.

To further test the moderating role of affective commitment in the relationship between job satisfaction and work engagement, we divided affective commitment into high and low groups by adding or subtracting a standard deviation from the mean and conducted a simple slope test (Figure 3). Results showed that, under the different conditions (high, medium, and low) of affective commitment, job satisfaction had a significant correlation with work engagement (bsimple slope=0.36, *t*=3.92, *p*<0.001; bsimple slope=0.50, *t*=6.74, *p*<0.001; bsimple slope=0.65, *t*=6.98, *p*<0.001, respectively). Moreover, bias-corrected percentile bootstrap analyses further showed that the indirect effect of work-family conflict on work engagement *via* job satisfaction was moderated by affective commitment. Specifically, for primary and secondary school principals who expressed high, medium, and low levels of affective commitment, the conditional indirect effect between work-family conflict and work engagement was significant [effect=−0.05, 95% CI=(−0.09, −0.02); effect=−0.07, 95% CI=(−0.12, −0.03); effect=−0.10, 95% CI=(−0.16, −0.04), respectively; see Table 5]. In sum, these results suggested that affective commitment moderated the relationship between work-family conflict and work engagement *via* job satisfaction, partially supporting hypothesis 4.

**Table 5:** Conditional indirect effect of affective commitment when job satisfaction mediated between work-family conflict and work engagement.

Mediator	Affective commitment	Effect	BootSE	95% CI
Job satisfaction	−1SD	−0.10	0.03	[−0.16, −0.04]
	M	−0.07	0.02	[−0.12, −0.03]
	+1SD	−0.05	0.02	[−0.10, −0.02]

## Discussion

The study mainly examined the influencing path of work-family conflict on work engagement among primary and secondary school principals, as well as the role of job satisfaction and affective commitment in the relationship between work-family conflict and work engagement. The results indicated that primary and secondary school principals’ work-family conflict was negatively correlated with their work engagement, and job satisfaction partially mediated the negative relationship between work-family conflict and work engagement. Furthermore, the relationship between job satisfaction and work engagement was moderated by affective commitment.

### Theoretical Implications

First, the study contributes to the literature on work-family conflict and work engagement. The results showed that primary and secondary school principals’ work-family conflict was negatively correlated with work engagement. This was consistent with previous studies (Sun et al., 2011; Simbula et al., 2012; Buonocore and Russo, 2013; Shao et al., 2015; Karatepe and Karadas, 2016; Wan et al., 2016; Lang et al., 2019; Liu and You, 2019; Zalewska, 2020). There was a negative correlation between work-family conflict and work engagement, and if conflict often occurs between work and family, engagement in work will be reduced. Moreover, this result also supports the conservation of resources theory and the role conflict theory. According to the conservation of resources theory, when individuals perceive or face actual resource loss and threat, they will reduce their efforts and avoid work in order to reduce loss (Hobfoll, 1989). Primary and secondary school principals need more resources and bear greater work pressures than ordinary teachers because they are not only managers and teachers but also the core of the school. Work-family conflict is more likely to occur when resources are insufficient, which reduces the degree of work engagement. Meanwhile, according to the role conflict theory (Linzer et al., 2002), primary and secondary school principals play multiple roles (such as teachers, managers, and family members), and conflict between work and family are more likely to occur, which may influence their work attitudes and behaviors.

Second, the study provides empirical evidence that may reveal the “black box” between work-family conflict and work engagement (Liu and You, 2019), and provides a new perspective to understand how and why work-family conflict is related to primary and secondary school principals’ work engagement. The results showed that job satisfaction plays a partially mediating role in the relationship between work-family conflict and work engagement, which strongly supports the mediating role of job satisfaction. Many studies have found that job satisfaction plays a mediating role in the relationship between work-family conflict and its outcome variables (e.g., job performance and turnover intention; Aminah, 2008; Chen et al., 2014; Ma et al., 2017). This suggested that primary and secondary school principals’ work-family conflict directly related to work engagement and also indirectly related to work engagement through job satisfaction. Meanwhile, the results also support those of previous studies reporting that work-family conflict is an important factor affecting teachers’ job satisfaction (Erdamar and Demirel, 2016; Ulucan, 2017), and job satisfaction was positively correlated with work engagement (Taghipour and Dezfuli, 2013; Huang et al., 2016; Wang et al., 2017; Park and Johnson, 2019). Following the conservation of resources theory, when individuals devote resources to coping with the conflict between work and family, then the resources available for other things will decrease. If the resources used for work are insufficient, work attitude and work efficiency will be affected, thus reducing job satisfaction. Moreover, if people’s satisfaction with their work declines, they may become passive toward their work, leading to a decrease in their work engagement. Conversely, if people feel positive about their job and satisfied with every aspect of work (such as pay, supervision, and workload), they are able to stay more motivated and committed to helping the company achieve its objectives and finally experience a desire to be involved and engaged in their work (Taghipour and Dezfuli, 2013). Primary and secondary school principals play multiple roles, which consumes a lot of energy. If there is conflict between work and family, it will lead to the increase of negative emotional experience and psychological burden of primary and secondary school principals, which will reduce their job satisfaction.

Finally, the study expands the research of the relationship among work-family conflict, job satisfaction, and work engagement in primary and secondary school principals. The results showed that affective commitment can moderate the positive relationship of job satisfaction and work engagement, specifically that the relationship between primary and secondary school principals’ job satisfaction and work engagement was significant under high and low levels of affective commitment. This is somewhat similar to the results of previous studies. For example, previous studies indicated that affective commitment can moderate the relationship between job resources and engagement (Dominguez et al., 2020), and the relationship between satisfaction with coworkers and supervisors, and lower turnover intention (Romeo et al., 2020). It can be seen that the higher the level of affective commitment, there is more work engagement and less turnover intention. Individuals with a high level of commitment to their organization may change the level of such attributions and continue to engage in their work (Namasivayam and Zhao, 2007).

However, our results showed that the relationship between job satisfaction and work engagement was not stronger under the high affective commitment than that of low effective commitment. This seems to be inconsistent with the previous research results. Previous studies have shown that, compared with low affective commitment, the relationship between variables (e.g., job resource and engagement, satisfaction with co-workers and supervisors and turnover intention) was stronger under the high affective commitment (Dominguez et al., 2020; Romeo et al., 2020). This may be related to the complex role of high affective commitment in the relationship among variables. A study found that under the condition of high-level effective commitment, the relationship between perceived organizational support and work engagement is U-shaped (Sun et al., 2015). For employees with high effective commitment, their definition of their roles is beyond the scope of their work, and they expect to perform duties outside their roles to repay their organization (Jiao et al., 2013). As a result, they will devote part of their work resources to their roles outside of work, and reduce the time and energy devoted to work (Sun et al., 2015). Primary and secondary school principals play multiple roles and bear more responsibilities. When their effective commitment level is high, they will repay the school through the responsibilities of other roles for the better development of the school, thus affecting their work engagement. Moreover, for individuals with high effective commitment, they have a higher degree of work identity and involvement. In this case, work engagement becomes a self-determination behavior, so the role of job satisfaction may be weak. In addition, for employees with low affective commitment, their role scope is relatively narrow, and the work role is regarded as the main responsibility (Sun et al., 2015). As the main way for them to invest in work, they are less willing to take on the outside responsibilities and role expectations (Sun et al., 2015). Therefore, for employees with low affective commitment, job satisfaction is closely related to work engagement.

### Practical Implications

The findings of this study have important practical implications. First, the study found that work-family conflict for primary and secondary school principals was negatively correlated with job satisfaction and work engagement. Therefore, the government and society should provide more opportunities for primary and secondary school principals to learn and train. This will not only improve principals’ work efficiency but will also reduce the time required for work, leaving them more time to spend with their families, which would eventually reduce conflict between work and family and facilitate their engagement with work. Second, the study found that affective commitment can moderate the relationships among work-family conflict, job satisfaction, and work engagement. Primary and secondary school principals with affective commitment tend to have a stronger sense of identity and belonging when their experiences within a school are consistent with their expectation, which enhances their sense of accomplishment and self-fulfillment and improves their work performance. Schools should focus on the cultivation of principals’ affective commitment to the management process, which plays an important role in improving principals’ work engagement and the development of the school. Finally, the study also found that job satisfaction was positively correlated with work engagement. Hence, we believe that the government and schools should provide good working conditions and give more encouragement and concern for primary and secondary school principals, which will help to improve their job satisfaction level and lead to greater work engagement.

### Limitations and Future Research

Although we verified the research hypotheses and provided an understanding of work-family conflict and work engagement, our study had some limitations. First, the cross-sectional nature of the present study could not confirm causality between the variables. Therefore, in order to reveal causal relationships more clearly, a longitudinal study design should be adopted in future research. Second, the relationship between work-family conflict and work engagement is complex, and there may be other mediating and moderating variables we did not examine. For example, one study found that emotional intelligence was closely related to work engagement (Brunetto et al., 2012). As a psychological trait, emotional intelligence is an affective psychological resource for coping with work pressure and negative situations, which can help individuals relieve pressure and reduce the consumption of internal resources (Karim and Weisz, 2011). Individuals with high emotional intelligence tend to experience less stress than individuals with low emotional intelligence (Kalyoncu et al., 2012). This suggests that emotional intelligence may buffer the negative relationship between work-family conflict and work engagement. Hence, future research should consider the role of other variables in order to better reveal the mechanism between work-family conflict and work engagement. Third, this study obtained the research data through self-report, although the common method deviation test results showed that the common method deviation problem was not considered to be great. Future studies can combine data from other sources (such as supervisors, colleagues, and family members) to obtain more objective results. Finally, the sample only contains China, which is not conducive to the promotion of the results. Future research should consider the role of cultural differences and conduct cross-cultural research to test the model of this study.

## Data Availability Statement

The datasets generated for this study are available on request to the corresponding author.

## Ethics Statement

The studies involving human participants were reviewed and approved by the morality and ethics committee of the School of Psychology, Guizhou Normal University. The participants provided oral informed consent to participate in this study.

## Author Contributions

ZY, SQ, LZ, and YP: conceptualization, funding acquisition, and project administration. ZY, LZ, and XH: data collection. ZY: formal analysis and writing of the original draft. ZY, SQ, LZ, XH, and YP: methodology. All authors contributed to the article and approved the submitted version.

### Conflict of Interest

The authors declare that the research was conducted in the absence of any commercial or financial relationships that could be construed as a potential conflict of interest.
